# Association of Age-Related Macular Degeneration with Erythrocyte Antioxidant Enzymes Activity and Serum Total Antioxidant Status

**DOI:** 10.1155/2015/804054

**Published:** 2015-02-28

**Authors:** Ivna Plestina-Borjan, Damir Katusic, Maria Medvidovic-Grubisic, Daniela Supe-Domic, Kajo Bucan, Leida Tandara, Veljko Rogosic

**Affiliations:** ^1^Department of Ophthalmology, University of Split School of Medicine, Spinciceva 1, 21000 Split, Croatia; ^2^Department of Ophthalmology, University of Zagreb School of Medicine, Salata 3, 10000 Zagreb, Croatia; ^3^Department of Ophthalmology, Institute of Navy Medicine, Soltanska 1, 21000 Split, Croatia; ^4^Department of Medical Laboratory Diagnostics, University of Split School of Medicine, Spinciceva 1, 21000 Split, Croatia

## Abstract

The aim was to estimate association of the oxidative stress with the occurrence of age-related macular degeneration (AMD). The activities of erythrocyte antioxidant enzymes: superoxide dismutase (SOD), glutathione peroxidase (GPx) and catalase (CAT) and additionally serum total antioxidant status (TAS) were used as indicators of the oxidative stress level. 57 AMD patients (32 early and 25 late AMD) and 50 healthy, age and gender matched controls were included. GPx activity (*P* < 0.001) and serum TAS (*P* = 0.015) were significantly lower in AMD patients. The difference was not significant for SOD or CAT activities. Significant interaction between GPx and SOD was detected (*P* = 0.003). At high levels of SOD activity (over 75th percentile), one standard deviation decrease in GPx increases the odds for AMD for six times (OR = 6.22; *P* < 0.001). ROC analysis revealed that combined values of GPx activity and TAS are significant determinants of AMD status. Accuracy, sensitivity, specificity, and positive and negative predictive values were 75%, 95%, 52%, 69%, and 90%, respectively. The study showed that low GPx activity and TAS are associated with AMD. SOD modulates the association of GPx and AMD. The results suggest that erythrocyte antioxidant enzymes activity and serum TAS could be promising markers for the prediction of AMD.

## 1. Introduction

Age-related macular degeneration (AMD) is the leading cause of legal blindness among people over 55 years in the Western countries and the third cause of blindness globally [1, 2]. It is a progressive, binocular disorder that affects nearly 20% of the population between 65 and 75 years of age and 35% over the age of 75 [3, 4]. According to the latest data from the World Health Organization (WHO), 14 million people worldwide are blind or severely visually impaired due to AMD [1]. These numbers are especially alarming given the increasing proportion of elderly people in the population.

Despite the severity of the problem, the etiology and pathogenesis of AMD are poorly understood and today's treatment possibilities are not satisfactory. Current therapy partially limits the damage only when it has already occurred but only in 5% of all the cases [5]. There are no any available treatments for dry form, which accounts 90% of AMD cases.

It is generally believed that AMD is caused by numerous biochemical, immunogenic, and environmental factors [6–8]. The most recent studies point to the key role of oxidative stress in the pathogenesis of AMD [4, 6–10]. Since oxidative stress involves almost all other assumptive pathogeneses and almost all risk factors for AMD, it could be crucial for the initiation and progression of the disease. Excessive generation of free radicals and other reactive oxygen species (ROS) and imbalance between their generation and the possibility of their degradation by the antioxidant defense system seem to be the most responsible factor in the development of AMD [10, 11].

ROS are generated continuously as a part of normal aerobic life as a byproduct of normal cellular metabolism (mitochondrial transport chain) [11] and additionally in the retina as the product of photochemical reaction between light and oxygen [12–14].

The retina, particularly macula, is the ideal environment for the generation of ROS due to the high oxygen consummation (because of its high metabolic activity) [15], lifelong exposure to light irradiation [16], high concentration of polyunsaturated fatty acids (PUFAs) [10], and abundance of photosensitizers [17, 18] in photoreceptors and RPE cells.

The consequences of oxidative damage on photoreceptors and RPE cells are severe because they are nonreplicating (postmitotic) cells and must survive a lifetime of oxidative insults [9].

The disorder occurs when the antioxidant system can no longer compensate the cumulative oxidative damage. The retina possesses a substantial number of antioxidants in the photoreceptor and RPE cells (especially in the area of the macula) [10]. Antioxidant defense includes enzymes: superoxide dismutase (SOD), glutathione peroxidase (GPx), and catalase (CAT); nonenzymatic antioxidants (as glutathione, uric acid, albumin, and bilirubin); and the antioxidant micronutrients (vitamin C, vitamin E, and carotenoids) [11, 19]. Antioxidant enzymes, which are of endogenous origin and constitute the first line of antioxidant defense, provide a more objective antioxidant state [10, 11, 19] than antioxidant micronutrients which depends on the current intake and does not indicate the real condition of the long-term defense against oxidative stress [19]. Antioxidant enzymes (SOD, CAT, and GPx) play the vital role in protecting the photoreceptors and RPE cells from oxidative damage [10, 20].

Hypothesis of oxidative stress induced AMD is supported by numerous animal, tissue cultures, or the donors (postmortem) retinas experiments [20–22] but not by clinical and epidemiological studies, which are less frequent and often contradictory [8, 23]. Direct estimation of blood oxidant levels is difficult because of very short free radicals half-life. However, oxidative stress can be estimated by measuring the antioxidant enzymes blood levels or activity. The greatest challenge is the development of the blood test that would identify individuals most at risk of developing AMD before any signs of the disease become apparent.

We hypothesized that low values of erythrocyte antioxidant enzymes activity and serum total antioxidant status (TAS) are associated with AMD. Our second hypothesis was that the interaction between antioxidant enzymes activities (SOD and GPX and/or SOD and CAT) could be of the same (or even larger) importance for AMD occurrence as low enzymes activity values are, because the combined action of the three enzymes forms one metabolic pathway for the protection against the oxidative damage [11]. Based on these hypotheses, we predicted that the evaluation of erythrocyte antioxidant enzymes activity and their interaction and serum TAS level measurement could be useful markers in the identification and selection of AMD predisposed individuals. Because the estimation of antioxidant enzymes in retinal cells* in vivo* is not possible, estimating their activity in peripheral blood would be a considerable advantage in the prediction of AMD occurrence, assuming that the obtained values correspond to the levels in the retinal cells.

## 2. Methods

The study was reviewed and approved by the Ethics Committee of the School of Medicine University of Split, Croatia, and performed in accordance with the ethical standards in the Declaration of Helsinki [24]. A written informed consent was obtained from each participant in the study.

In total, the study included 107 subjects aged 60 years or older: 57 of them with AMD (25 with late and 32 with early AMD) and 50 age and gender matched controls without apparent AMD-related fundus.

The participants in the study were recruited from the regular outpatients of the Eye Clinic (Retinal Department) at the University Hospital in Split, during the period from November 2012 to December 2013. Subjects with diabetes, verified cardiovascular disease patients, cancer patients, smokers, alcoholics, and patients with advanced cataract or other disturbances at the anterior segment of the eye that prevents a detailed fundus examination as well as patients with glaucoma were excluded. Patients under or after anti-VEGF treatment were not included in the study. Patients taking vitamins with antioxidant effect were asked to stop taking them for 2 months. Later on, they were included in the study if they met the criteria. All respondents taking medications that can lower the activity of antioxidant enzymes (e.g., large doses of paracetamol and some cytostatics) were excluded from the research.

A comprehensive ophthalmologic examination of all subjects was done. The best corrected visual acuity (although it was not included in the classification of disease) was determined according to the Snellen optotypes. A detailed general ophthalmologic examination (anterior segment slit lamp examination and intraocular pressure measurement) was done. AMD was diagnosed by slit lamp biomicroscopy of the fundus with the Maistner WF contact lens. In all AMD patients, the color fundus photography was taken and fluorescein angiography (FA) was done on digital fundus camera Zeiss FF 450 plus IR. In the control group, only color fundus photographs were taken and FA was not done, as well as in the AMD patients with allergy history. All participants also underwent optical coherence tomography (OCT), on Cirrus HD-OCT Model 400, Carl Zeiss Meditec.

Presence and severity of AMD were determined from fundus photographs and FA and graded according to the AMD International Classification and Grading System [25]. The patients were classified according to the findings in the more affected eye, in the case of bilateral affection. Early form of AMD includes small and large soft drusen (≥63 *μ*m) as well as confluent drusen and areas of hyperpigmentation and hypopigmentation with no visible choroidal blood vessels within 3000 *μ*m of foveola. Late form of AMD includes wet (neovascular) and dry (geographic atrophy) form in the area within 3000 *μ*m from foveola. Neovascular form is defined by the presence of serous or hemorrhagic RPE or sensory retinal detachment, subretinal neovascularization, and fibrovascular scar. Geographic atrophy implies sharply limited areas of depigmentation, greater than 175 *μ*m with visible choroidal vascularization.

### 2.1. Blood Samples and Enzyme Assays

A sample of venous blood (4 mL) was taken from all the subjects, between 8 and 9 a.m., after 12 to 14 hours of overnight fasting, in order to determine the antioxidant erythrocyte enzymes (SOD, GPx, and CAT) activity and TAS in serum. The blood sample for SOD and CAT was drawn into vacuum tubes (Becton Dickinson) with the addition of ethylenediaminetetraacetic acid (K3-EDTA) as anticoagulant. For GPx vacuum tubes (Becton Dickinson) were used with the addition of lithium heparin. Within 1 hour of blood samples collection, enzymes activity was measured in erythrocyte lysate spectroscopically at the temperature of 37°C. For assessing the TAS, blood was taken in siliconized vacuum tubes (Becton Dickinson) without anticoagulant.

The commercial RANSOD assay (Randox Laboratories, UK) was used to quantify the SOD activity in erythrocyte lysate. After isolating them from the whole blood, the erythrocytes were lysed following the manufacturer's instructions (procedure applied by Winterbourn et al. [26] with minor modifications). The whole fresh blood sample was centrifuged at 3000 rpm for 10 minutes. Immediately after this procedure, plasma with leukocytes and platelets was carefully removed. Erythrocytes were washed 4 times with 0.9% saline NaCl and centrifuged 10 minutes at 3000 rpm after each wash. Subsequently they were mixed with cold distilled water and left for 15 minutes at 4°C to complete the process of hemolysis. Lysate was then diluted with 0,01 mol/L phosphate buffer pH 7.0 (0.05 mL lysate + 1200 mL phosphate buffer or 50 *µ*L lysate with 1.2 mL of phosphate buffer). SOD activity was determined spectrophotometrically at a wavelength of 505 nm and the temperature of 37°C in the diluted lysate and expressed in U/g Hb.

Hemoglobin was measured in the whole blood sample by standard laboratory method.

GPx activity was measured in the sample of whole blood hemolysate by the commercial RANSEL assay (Randox Laboratories, Crumlin, UK), according to the Paglia and Valentine method [27] and following the manufacturer's instructions. Heparinized blood (0.05 mL) was diluted with 1 mL of the diluting agent (R 3, included in kit) and incubated for 5 minutes. After that 1 mL Drabkin's reagent was added and mixed thoroughly. Within 20 minutes GPx activity was assessed at 340 nm and 37°C and expressed in U/g Hb.

SOD and GPx measurements were done on automatic analyzer Architect C8000 (Abbott Diagnostic, USA).

Catalase activity was also determined in erythrocyte lysate using the OxiSelect catalase activity assay (Cell Biolabs, San Diego, CA, USA). The assay was performed following the manufacturer's assay procedure. The CAT degrades H_2_O_2_ to water and molecular oxygen, and the amount of degraded H_2_O_2_ is proportional to the enzyme activity. The color change of the reaction mixture was measured spectrophotometrically at 520 nm, on the BioTek analyzer (MTX Lab Systems, LLC. Virginia, USA). CAT activity was calculated using a calibration curve and expressed as U/g Hb.

TAS was determined in serum using the commercially available TAS kit (Randox, Laboratories, Crumlin, UK) following the manufacturer's instruction by the method reported by Miller et al. [28] ABTS [2,2′-azinobis(3-ethylbenzothiazoline-6-sulfonate)] incubated with peroxidase (metmyoglobin) and hydrogen peroxide (H_2_O_2_) produces free radical cation ABTS•+, green in color (detected on 600 nm). Antioxidants present in the added serum sample suppress the formation of the color. The concentration of antioxidants is inversely proportional to the development of the color. Concentration of TAS is expressed as mmol/L of Trolox equivalents. TAS was also measured on automatic analyzer Architect C8000.

### 2.2. Statistical Procedures

The level of statistical significance was set to *P* < 0.05 and all confidence intervals were given at 95% level. In all instances, two-tailed tests of statistical significance were used. The univariate analysis of differences in median values of TAS, GPx, SOD, and CAT between AMD and control group was done by Mann-Whitney *U* test with Monte Carlo two-tailed statistical significance based on 10.000 sampled tables. The distributions were described by medians and interquartile ranges. The absolute and relative differences between medians in AMD and in control group were reported. The confidence intervals (CI) of the difference of two medians were calculated based on the method proposed by Bonett and Price [29]. The multivariate logistic regression analysis was used to estimate enzymes activity as independent predictors of the occurrence of AMD and the interaction between GPx and SOD activities. All markers were standardized before the analysis and expressed as *z* values. The standardization was done by subtracting arithmetic means from all data and dividing the differences by standard deviation of each marker. The moderating effect of SOD on association of GPx activity and AMD was analyzed by “Process,” release 2.12, Andrew F. Hayes, the Ohio State University, 2014. SOD value defining the region of statistically significant association of GPx and AMD was assessed by Johnson-Neyman technique as implemented in the “Process.” The probabilities of AMD were calculated from odds as follows: probability = odds ratio/(1 + odds ratio). The optimal cut-off points of TAS and GPx for the prediction of AMD were determined by Receiver Operating Characteristic (ROC) analysis and Youden index J. Two markers were dichotomized and combined into the composite predictor with two values: (1) at least one of two indices bellow the cut-off value and (2) both TAS and GPx above the cut-off values. The diagnostic accuracy of the combined predictor was accessed by the area under the curve, sensitivity, specificity, positive and negative predictive values, and likelihood ratios. Data analysis was done by R Development Core Team (2008) (R: a language and environment for statistical computing), R Foundation for Statistical Computing, Vienna, Austria, ISBN 3-900051-07-0, URL http://www.R-project.org/. ROC curve analysis was done by MedCalc Statistical Software version 13.3 (MedCalc Software bvba, Ostend, Belgium; http://www.medcalc.org/; 2014).

## 3. Results

A total of 107 subjects were enrolled in this study: 57 patients in AMD group (25 with late and 32 with an early form of AMD) and 50 in control group. The groups were properly matched by age and gender. In the AMD group of patients, there were 36 (61.4%) female and 21 (38.6%) male, while in the control group there were 31 (62%) female and 19 (38%) male. In the AMD group of patients, the median of age was 77 years (interquartile range: 71–82 years) while the median of age in the control group was 77.5 years (interquartile range: 73–81).

GPx activity was significantly lower in subjects with AMD compared to controls by 15 U/g Hb (*P* < 0.001) (Table 1). Likewise, median of TAS value was 0.17 mmol/L lower in AMD group than in the control group, indicating statistically significant difference between these two groups (*P* = 0.015). When adjusted for the effects of all four antioxidant indicators, decrease of GPx and TAS has shown significant association with AMD. No statistically significant differences in activity of erythrocyte SOD (*P* = 0.984) and CAT (*P* = 0.426) were observed between AMD patients and controls.

**Figure 1:**
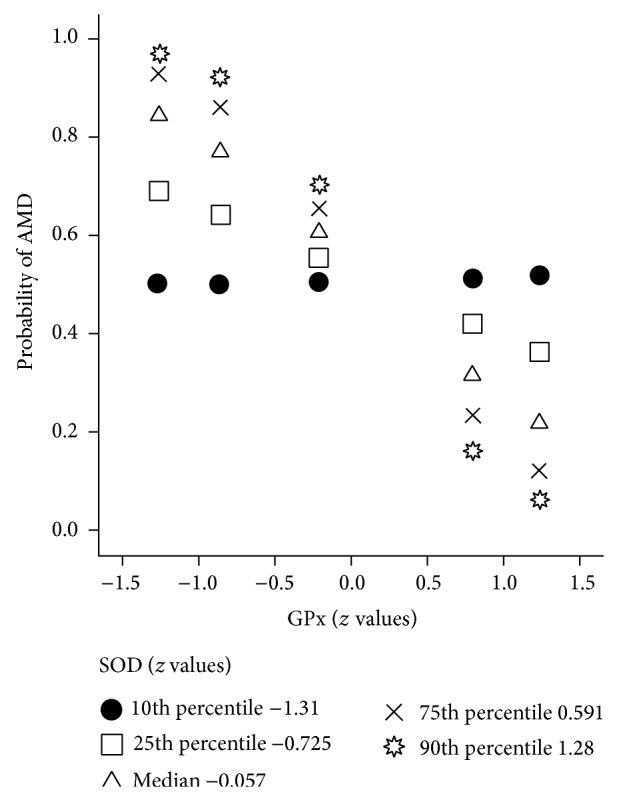
Probability of age-related macular degeneration (AMD) predicted by glutathione peroxidase (GPx) activity values at different levels of superoxide dismutase (SOD) activity; all values are standardized and expressed as *z* values; arithmetic mean was subtracted and the difference was divided by standard deviations.

Although SOD activity has not been significantly associated with AMD, it had statistically significant moderating effect on the association of GPx and AMD (*P* = 0.003). At SOD activity value lower than 1st quartile that is lower than −0.73 standard deviations (corresponding to 1209 U/g Hb), differences in GPx activity were not significantly associated with AMD (Table 2). Above 75th percentile of SOD values, which is higher than 0.59 standard deviations (corresponding to 1581 U/g Hb), one standard deviation decrease in GPx increases the odds for AMD for six times (OR = 6.22; *P* < 0.001). The higher the value of SOD activity is, the higher the association of decrease in GPx activity with AMD is (Figure 1).

Interactions between SOD and CAT (*P* = 0.810) and GPx and CAT (*P* = 0.679) were not statistically significant.

The ROC analysis was performed in order to investigate the potential of combined GPx activity and TAS levels as clinical tool for the prediction of AMD status (healthy versus diseased) and to identify the threshold levels of GPx activity and TAS that discriminate well patients from the healthy subjects with the maximized sensitivity. Pretest probability of disease or the expected prevalence was set to 25% as the average prevalence in the population ≥65 years of age [2, 4]. Optimal cut-off values determined by Youden index *J* were 1.44 mmol/L for TAS and 48.97 U/g Hb for GPx.

Two markers were combined into the final predictor with two values: (1) at least one of two indices bellow the cut-off value and (2) both TAS and GPx above the cut-off values. Total accuracy, measured by area under the curve (AUC), of the classification of patients into AMD or control group was AUC = 75% (95% CI, 66%–79%; *P* < 0.001), with sensitivity of 95% and specificity of 52% (Table 3). Positive likelihood ratio for AMD was 1.97 (95% CI, 1.52–2.26). Negative likelihood ratio was 0.10 (95% CI, 0.03–0.31). Number needed to diagnose was 2.1 (95% CI, 1.82–3.37).

## 4. Discussion

The goal of the present study was to investigate the hypothesis that erythrocyte antioxidant enzymes activity (SOD, GPX, and CAT) and the serum TAS are associated with the pathogenesis of AMD.

No significant difference was observed in erythrocyte SOD and CAT activity between AMD patients and controls in our study, while the GPx activity and TAS level were significantly lower in the patients with AMD compared to the controls. Moreover, significant interaction between GPx and SOD was detected. At very low SOD activity values decrease in GPx activity was not associated with AMD, but at high SOD values decrease in GPx significantly increased the odds for AMD (more than six times).

Previously published research data, based mainly on examining the levels or the activity of the antioxidant enzymes in donor retinal tissues [30], tissue cultures [31], or animal models [22, 32], as well as the results of few clinical studies [8, 33–35], seem to be in contradiction. It has been proposed that antioxidant enzymes are lower in AMD patients contributing to the development of the disease. We assume that also higher SOD accompanied by reduced GPx and reduced or unchanged CAT activity (as shown in our study) contributes significantly to the formation of the toxic level of hydrogen peroxide (H_2_O_2_) and to an increased oxidative damage. Removal of excess of superoxide anions by SOD is an important antioxidant defense mechanism, although too much SOD (in relation to the activities of H_2_O_2_ removing enzymes: GPx and CAT) may be deleterious. H_2_O_2_ is itself ROS and can be converted into a more reactive and damaging hydroxyl radical (OH^•^) (via Fenton reaction), which readily initiates lipid peroxidation chain reaction. In our opinion, lipid peroxidation is the main oxidative mechanism for damaging photoreceptor outer segment lipid membranes (rich with PUFAs), RPE cell, and organelles membranes (especially mitochondrial) [11, 36], leading to the development of AMD. Therefore, SOD acts in cooperation with GPx and CAT which convert H_2_O_2_ to nontoxic products: water and molecular oxygen, protecting the photoreceptors and RPE cells from oxidative damage. GPx is more effective than CAT in detoxification of H_2_O_2_ because of its kinetic properties [37]. Other than on H_2_O_2_, GPx acts on lipid hydroperoxides and on lipid peroxidation-derived toxic aldehydes, reducing them to corresponding alcohols [11].

The decrease in GPx activity can result from the increased enzyme consumption caused by a higher level of oxidative stress or from the deficiency of essential metal cofactors (selenium). On the other hand, it can be a consequence of the decreased expression of genes that regulate enzymes production or the formation of inactive enzyme complexes (because of genetic variants of enzymes with variable activities) [11, 37].

Animal studies support our results indicating that low values of GPx contribute to the development of AMD [22, 38]. Contrary to our results, the Pathologies Oculaires Liées à l'Age (POLA) study [8] found that higher levels of plasma GPx were associated with a ninefold increase in late AMD prevalence. In POLA study, GPx plasma levels were measured, not activities. Measuring the levels of antioxidant enzymes is less reliable than the determination of their activities. Higher levels do not always mean higher activities due to genetic-based creation of inactive enzyme complex [11]. There is also no information about the interaction between GPx and other antioxidant enzymes in POLA study.

To our knowledge, our study is the first to examine the interaction between the antioxidant enzymes activity ratio in AMD patients compared to healthy controls.

The study indicates that increased SOD activity at low GPx activity (as well as lack between SOD and CAT interaction) significantly increases the probability for AMD. It could be crucial in explaining the impact of antioxidant enzyme activity in the occurrence of AMD in our patients (stressing the impact of lipid peroxidation).

Several experimental studies have provided a solid background for our research [11, 39]. The antioxidant defense operates as a balanced and coordinated system (SOD and GPx and SOD and CAT). Previous clinical studies on the impact of antioxidant enzymes on AMD, differently conceived from our study, provide contradictory data, because they did not examine interaction of enzyme activities in association with AMD, as we did [8, 33, 34]. Some of them measured serum or erythrocytes level (not activity) of antioxidant enzymes [8, 34] and others measured only particular antioxidant enzymes (not all three) with [35, 40] or without TAS levels [33, 34, 37].

In the present study the level of serum TAS (representing also serum nonenzymatic antioxidants) was found to be significantly lower in patients with AMD compared to controls. These results are consistent with other studies [35, 40] that all associate lower serum TAS level with a higher risk of AMD development.

The method of measuring serum TAS levels by Randox commercial TAS kit, as well as other methods (Ferric-reducing antioxidant power assay, oxygen radical absorbing capacity, etc.) [11], has certain limits. One of the main disadvantages of this method is that sample diluting might result in false positive TAS values, but that was not required in our research because in none of the samples TAS values were above 2.5 mmol/L [41]. In addition, control serum was assayed in each batch of samples for the estimation of analytical imprecision.

We included serum TAS in our study because of synergistic and compensatory effect between the intracellular antioxidant enzymes and extracellular nonenzymatic antioxidants in minimizing oxidative damage [11]. Our results indicate lack of compensatory TAS effect in lowering tissue damage in AMD patients contrary to the findings in the control group of subjects.

We have also tried to predict AMD occurrence based on the combined optimal cut-off values of erythrocyte GPx activity and serum TAS level determined by ROC analysis with the maximized test sensitivity. The ROC analysis showed that combined values of these two markers could provide promising predictive model for AMD (with 75% of AUC and 95% of the test sensitivity).

The main limitation of this study was a relatively small sample size. In addition, our predictive model has not been evaluated on the independent sample (that is our next step), but we think that this limitation did not affect our results significantly.

In conclusion, we can say it is likely that low peripheral blood antioxidant enzymes activity well reflects retinal cells antioxidant status and could contribute to the occurrence of AMD. Assessment of antioxidant erythrocyte enzyme activity and its interaction combined with serum TAS measurement could be a promising marker in predicting the occurrence of AMD.

The antioxidant mechanism is not completely clear. There are still many open questions to be answered; for example, can the overexpression of antioxidant enzymes, which generally provides protection against oxidative stress in cell culture models, be applied to the whole organism, or to what extent does the overexpression of antioxidant enzymes provide protection, or how to reestablish antioxidant enzymes activity balance? Our results demonstrate the need for future genetic, biochemical, and biomolecular research.

## Figures and Tables

**Table 1:** Difference of median erythrocyte SOD, GPx, and CAT activities and serum TAS between AMD patients and healthy control group; association of decrease in markers activity with AMD.

	AMD	Control	*d*_abs_ (95% CI)	*d*_*rel*⁡_	*P*	Multivariate^*^	
	(*n* = 57)	(*n* = 50)				OR (95% CI)	*P*
TAS mmol/L	1.47 (1.33–1.71)	1.64 (1.47–1.77)	−0.17 (−0.28–−0.05)	10%	0.015	2.34 (1.42–3.86)	0.001
GPx U/gHb	39 (33–48)	54 (43–62)	−15 (−21–−9)	28%	<0.001	4.10 (2.19–7.70)	<0.001
SOD U/gHb	1389 (1185–1601)	1413 (1221–1575)	−25 (−166–116)	2%	0.984	1.10 (0.68–1.79)	0.701
CAT U/gHb	52 (48–58)	54 (47–60)	−2 (−6–2)	4%	0.426	0.70 (0.41–1.20)	0.191

Values are presented as median (interquartile range).

AMD = age-related macular degeneration; TAS = serum total antioxidant status index; GPx = glutathione peroxidase; SOD = superoxide dismutase; CAT = catalase.

*P* = Mann-Whitney *U* test with Monte Carlo two-tailed statistical significance based on 10.000 sampled tables; *d*_abs_ = absolute difference between medians; *d*_*rel*⁡_ = relative difference between medians; 95% CI = 95% confidence interval of difference between medians; OR = odds ratio for AMD at unit decrease of standardized markers' values, multivariate (adjusted) binary logistic regression.

^*^Values of TAS, GPx, SOD, and CAT were standardized before the multivariate logistic regression analysis; arithmetic mean was subtracted and the difference was divided by standard deviation.

**Table 2:** Conditional effect of decrease in GPx activity on AMD at different levels of SOD.

SOD^*^ (*z* values)	Effect of decrease of GPx			
	*B* (95% CI)	*P*	OR	Probability of AMD
10th percentile (−1.31)	−0.02 (−0.79–0.74)	0.953	0.98	0.49
25th percentile (−0.73)	0.55 (0.00–1.09)	0.049	1.73	0.63
50th percentile (−0.06)	1.20 (0.63–1.76)	<0.001	3.31	0.77
75th percentile (0.59)	1.83 (0.98–2.67)	<0.001	6.22	0.86
90th percentile (1.28)	2.50 (1.27–3.73)	<0.001	12.16	0.92

GPx = glutathione peroxidase; SOD = superoxide dismutase; *B* = regression coefficient; 95% CI = 95% confidence intervals; *P* = level of statistical significance; OR = odds ratio for AMD at unit decrease in standardized GPx values; probability = probability of AMD predicted by logistic regression of decrease of GPx *z* values.

^*^Values of GPx, SOD, and CAT were standardized before the logistic regression analysis and expressed as *z* values; arithmetic mean was subtracted and the difference was divided by standard deviation.

**Table 3:** Prediction of AMD based on the combined erythrocyte GPx activity and serum TAS value.

	AMD (*n* = 57)	Control (*n* = 50)	Predictive value
At least one bellow the cut-off^*^	54 (94.7)	24 (48.0)	Positive 0.69 (0.63–0.72)
Both TAS and GPx above the cut-off	3 (5.3)	26 (52.0)	Negative 0.90 (0.74–0.97)
Total	**57 (100.0)**	**50 (100.0)**	

	Sensitivity 0.95 (0.87–0.99)	Specificity 0.52 (0.43–0.56)	

Values are presented as *n* (%).

AMD = age-related macular degeneration; TAS = serum total antioxidant status index; GPx = glutathione peroxidase.

^*^Cut-off values were set up based on the Youden index *J*, ≤1.44 for TAS, ≤48.97 for GPx.
